# Cyclic arginine-glycine-aspartate attenuates acute lung injury in mice after intestinal ischemia/reperfusion

**DOI:** 10.1186/cc12493

**Published:** 2013-01-29

**Authors:** Shingo Matsuo, Weng-Lang Yang, Monowar Aziz, Asha Jacob, Ping Wang

**Affiliations:** 1 Department of Surgery, Hofstra North Shore - LIJ School of Medicine and Laboratory of Surgical Research, The Feinstein Institute for Medical Research, 350 Community Drive, Manhasset, NY 11030, USA

## Abstract

**Introduction:**

Intestinal ischemia is a critical problem resulting in multiple organ failure and high mortality of 60 to 80%. Acute lung injury (ALI) is a common complication after intestinal ischemia/reperfusion (I/R) injuries and contributes to the high mortality rate. Moreover, activated neutrophil infiltration into the lungs is known to play a significant role in the progression of ALI. Integrin-mediated interaction is involved in neutrophil transmigration. Synthetic peptides containing an arginine-glycine-aspartate sequence compete with adhesive proteins and inhibit integrin-mediated interaction and signaling. Thus, we hypothesized that the administration of a cyclic arginine-glycine-aspartate peptide (cRGD) inhibited neutrophil infiltration and provided protection against ALI induced by intestinal I/R.

**Methods:**

Ischemia in adult male C57BL/6 mice was induced by fastening the superior mesenteric artery with 4-0 suture. Forty-five minutes later, the vascular suture was released to allow reperfusion. cRGD (5 mg/kg body weight) or normal saline (vehicle) was administered by intraperitoneal injection 1 hour prior to ischemia. Blood, gut, and lung tissues were collected 4 hours after reperfusion for various measurements.

**Results:**

Intestinal I/R caused severe widespread injury to the gut and lungs. Treatment with cRGD improved the integrity of microscopic structures in the gut and lungs, as judged by histological examination. Intestinal I/R induced the expression of β_1_, β_2 _and β_3 _integrins, intercellular adhesion molecule-1, and fibronectin. cRGD significantly inhibited myeloperoxidase activity in the gut and lungs, as well as neutrophils and macrophages infiltrating the lungs. cRGD reduced the levels of TNF-α and IL-6 in serum, in addition to IL-6 and macrophage inflammatory protein-2 in the gut and lungs. Furthermore, the number of TUNEL-staining cells and levels of cleaved caspase-3 in the lungs were significantly lowered in the cRGD-treated mice in comparison with the vehicle mice.

**Conclusions:**

Treatment with cRGD effectively protected ALI and gut injury, lowered neutrophil infiltration, suppressed inflammation, and inhibited lung apoptosis after intestinal I/R. Thus, there is potential for developing cRGD as a treatment for patients suffering from ALI caused by intestinal I/R.

## Introduction

Intestinal ischemia/reperfusion (I/R) injury is a critical problem resulting in high mortality rates of up to 60 to 80% [1-3]. I/R causes gut barrier disruption and endotoxin entry into the circulation, leading to severe systemic inflammation and eventually multiple organ failure [4-7]. Acute lung injury (ALI) is a common complication that occurs after intestinal I/R and is a major contributor to the high mortality rate [7-11]. Limited pharmacological treatment options exist for ALI, with most targeting inflammatory mediators and oxidative stress pathways [12]. There is an urgent need for an effective approach for ALI treatment.

ALI induced by intestinal I/R is caused by an excessive systemic inflammatory response, triggered by the release of proinflammatory cytokines and bacteria-derived endotoxins from the reperfused ischemic gut tissue [13-15]. Pathophysiologically, ALI is associated with the influx and activation of immune cells. With the release of abundant cytokines and chemokines, ALI can be further complicated by infection and ventilation-induced injury [13,16]. Neutrophils are the earliest immune cells to be recruited to the site of injury or infection. Moreover, recruitment of neutrophils to the lungs is known to play a key role in the progression of ALI [17]. However, neutrophil migration into the lungs is not sufficient to cause ALI, but rather activation of neutrophils is necessary to trigger the injury [17]. Under normal conditions, neutrophils roll along microvascular walls. After activation by proinflammatory cytokines and chemotactic factors, neutrophils are then able to penetrate the vasculature and transmigrate through the interstitium into the alveolar space [18,19]. In addition, alveolar macrophages are also essential players in the initiation of ALI [11,20].

Integrins expressing on the cell surface mediate the interactions of neutrophils with endothelial cells and extracellular matrix (ECM) proteins to strengthen adhesion and migration for complete infiltration [21,22]. Twenty-four different integrins are expressed in humans, each composed of noncovalently associated α and β chains [23]. Neutrophil adhesion is commonly mediated through β_2 _integrins to interact with adhesion molecules (for example, intercellular adhesion molecule (ICAM)-1, ICAM-2, and vascular cell adhesion molecule-1) on the endothelial surface [17]. Inhibition of β_2 _integrins attenuates neutrophil migration into the lungs [24]. In addition to β_2 _integrins, α_4_β_1 _and α_5_β_1 _integrins also contribute to neutrophil migration by interacting with vascular cell adhesion molecule-1 during pulmonary inflammation [25]. β_1 _and β_3 _integrins mediate the interaction with ECM proteins, such as collagen, laminin, vitronectin and fibronectin [26]. In addition, integrins are also associated with phagocytosis, reactive oxygen species and cytokine productions [27,28].

Through sequence analysis of ligands bound to integrins, a minimal recognition sequence in ligands containing amino acid sequence arginine-glycine-aspartate (RGD) has been well identified [26]. The RGD motif enables ligand binding to integrins and regulates cell growth, migration and survival [26]. In contrast, synthetic peptides containing the RGD sequence can compete with adhesive molecules for binding to integrins and disrupt the cellular activity mediated by integrin interaction. For example, a synthetic RGDS peptide has shown to attenuate lipopolysaccharide-induced pulmonary inflammation and to reduce the number of neutrophils infiltrating the lungs by inhibiting integrin-mediated mitogen-activated protein kinase activation [29]. Administration of cyclic RGD peptides inhibited the recruitment of macrophages and neutrophils, as well as depressing the expression of proinflammatory mediators in steatotic liver cold ischemia and reperfusion injury [30]. Another study demonstrated the effectiveness of cyclic RGD peptides in ameliorating ischemic acute renal failure in rats [31].

We have previously shown that treatment with milk fat globule-epidermal growth factor-factor 8, a protein containing an RGD motif, attenuates inflammation and ALI after intestinal I/R [6]. Based on our observations and other studies, we reasoned that RGD peptides have the capability of alleviating the injury caused by I/R. In this study, we treated mice with a cyclic RGD peptide (cRGD; RGDFV) that selectively interacts with β_3 _and β_5 _integrins [32]. We then evaluated its effectiveness on alleviating the ALI induced by intestinal I/R.

## Materials and methods

### Experimental model

Male C57BL/6 mice (20 to 25 g; Taconic, Albany, NY, USA) were used in all experiments. Animals were pre-medicated via intraperitoneal injection with either 0.2 ml of 5 mg/kg body weight (BW) cRGD (cyclo RGDfV; Enzo Life Sciences, Farmingdale, NY, USA) or normal saline (vehicle) 1 hour prior to surgery. Mice underwent induction of anesthesia with inhalational isoflurane. An upper midline laparotomy was performed to expose the abdomen and the superior mesenteric artery was occluded by fastening with 4-0 silk suture and a PE10 catheter (5 mm length). Cessation of blood flow to the intestines was judged by color changes to paleness of peripheral mesenteric vessels of the duodenum, jejunum and ileum. After 45 minutes of ischemia, the superior mesenteric artery suture was removed and mice were administered 0.5 ml sterile saline into the peritoneal cavity to improve dehydration after intestinal I/R. Sham animals underwent only midline laparatomy incision and closure, without intestinal ischemia or treatment. At 4 hours, blood, lung and gut tissues were collected. A section of each tissue sample was preserved in formalin for histopathology. Blood and the remainder of tissue samples were frozen immediately with liquid nitrogen, and stored at -80°C until measurements.

All experiments were performed in accordance with the guidelines for the use of experimental animals by the National Institutes of Health (Bethesda, MD, USA) and were approved by the Institutional Animal Care and Use Committee of The Feinstein Institute of Medical Research (Manhasset, NY, USA).

### Histopathological examination

Gut and lung tissues were fixed in 10% formalin and then embedded in paraffin. Tissue blocks were sectioned at a thickness of 5 μm, transferred to glass slides, and stained with H & E. Morphologic examination of these tissues was evaluated under a light microscope in a blinded manner. The severity of gut injury was scored from 0 to 4 by assessing villus-to-crypt ratio (normal ratio, 5:1), lymphocytic infiltrates, epithelial degeneration/necrosis, erosions, glandular dilatation, and transmural changes [33]. The severity of lung injury was scored from 0 to 4, based on the presence of exudates, hyperemia/congestion, neutrophilic infiltrates, intra-alveolar hemorrhage/debris, and cellular hyperplasia [34].

### Myeloperoxidase activity assay

Tissues of gut and lungs were homogenized in KPO_4 _buffer containing 0.5% hexa-decyl-trimethyl-ammonium bromide. After centrifugation the supernatant was diluted in reaction solution, and the rate of change in optimal density for 2 minutes was measured at 460 nm to calculate myeloperoxidase (MPO) activity [6].

### RT-PCR analysis

Total RNA was extracted from gut and lung tissues using a Trizol reagent (Invitrogen, Carlsbad, CA, USA) and was reverse-transcribed into cDNA using murine leukemia virus reverse transcriptase (Applied Biosystems, Foster City, CA, USA). A PCR reaction was carried out in 25 μl of a final volume containing 0.08 μmol of each forward and reverse primer, cDNA, and 12.5 μl SYBR Green PCR Master Mix (Applied Biosystems). Amplification was conducted in an Applied Biosystems 7300 real-time PCR machine under the thermal profile of 50°C for 2 minutes, 95°C for 10 minutes followed by 45 cycles of 95°C for 15 seconds and 60°C for 1 minute. The level of mouse β-actin mRNA was used for normalization. Relative expression of mRNA was expressed as the fold change in comparison with the sham tissues. The primers used for this study are listed in Table 1.

**Table 1:** Primer sequences used in this study

Gene	GenBank	Forward	Reverse
β_1 _integrin	NM_010578	AACTGCACCAGCCCATTTAG	ACATTCCTCCAGCCAATCAG
β_2 _integrin	NM_008404	GTGGTGCAGCTCATCAAGAA	TCGGAAGCCATGACCTTTAC
β_3 _integrin	NM_016780	GCTCATTGGCCTTGCTACTC	CCCGGTAGGTGATATTGGTG
ICAM-1	NM_010493	GGGCTGGCATTGTTCTCTAA	CTTCAGAGGCAGGAAACAGG
Fibronectin	NM_010233	GAGGAGGGAGATGAACCACA	GGGTCTACTCCACCGAACAA
β-actin	NM_031144	CGTGAAAAGATGACCCAGATCA	TGGTACGACCAGAGGCATACAG

ICAM-1, intercellular adhesion molecule-1.

### Immunofluorescence staining

Paraffin-embedded sections of gut and lung tissues were dewaxed in xylene and rehydrated in a graded series of ethanol. Slides were incubated in 0.92% citric acid buffer (Vector Laboratories, Burlingame, CA, USA) at 95°C for 15 minutes. After cooling to room temperature, the slides were incubated with 2% H_2_O_2 _in 60% methanol and blocked in 2% normal rabbit serum/Tris-buffered saline, after which they were incubated with an anti-Gr-1 antibody conjugated with biotin (BioLegend, San Diego, CA, USA), followed by streptavidin-FITC (BD Biosciences, Franklin Lakes, NJ, USA), or anti-CD11b antibody conjugated with PE (BD Biosciences) for 1 hour. Slides were counterstained with 4',6-diamidino-2-phenylindole and examined under a fluorescence microscope. Cells stained with anti-Gr-1 or CD11b antibodies were counted per 10 visual fields at 200× magnification.

### Measurements of cytokines and chemokines

IL-6 and TNF-α were quantified by using a specific mouse ELISA kit (BD Biosciences) in serum, gut and lung tissues. Macrophage inflammatory protein (MIP)-2 was measured using a mouse ELISA kit (R&D Systems, Minneapolis, MN, USA) in gut and lung tissues.

### Terminal deoxynucleotidyl transferase dUTP nick end-labeling assay

Lung histopathological slides were dewaxed and incubated with proteinase K. Slides were stained using a terminal deoxynucleotidyl transferase dUTP nick end-labeling (TUNEL) kit (Roche Diagnostics, Indianapolis, IN), counterstained with propidium iodide and examined under a fluorescence microscope. Apoptotic cells appeared green fluorescent and were counted per 10 visual fields at 200× magnification.

### Western blotting

Lung tissue was homogenized in lysis buffer (10 mM Tris-HCl, pH 7.5, 120 mM NaCl, 1% NP-40, 1% sodium deoxycholate, and 0.1% sodium dodecyl sulfate) containing a protease inhibitor cocktail (Roche Diagnostics) by sonication. Total lung lysate was fractionated on Bis-Tris gels (4-12%) and transferred to polyvinylidene difluoride membranes. The membranes were blocked by PBS (0.2×) with 0.1% casein and then incubated with anti-cleaved caspase-3 (Cell Signaling Technology, Beverly, MA, USA) or anti-β-actin (Sigma, St Louis, MO, USA) antibodies diluted in PBS (0.2×) with 0.1% casein and 0.1% Tween 20. After the wash, the membranes were incubated with fluorescence-labeled secondary antibodies and scanned by the Odyssey imaging system (LI-COR Biosciences, Lincoln, NE, USA). The band intensity was analyzed by the Odyssey densitometric software.

### Statistical analysis

Data are expressed as mean ± standard error and compared with one-way analysis of variance and the Student-Newman-Keuls test for multiple group analyses. Student's *t *test was applied for a pair comparison. Differences in values were considered significant if *P *< 0.05.

## Results

### cRGD attenuates gut damage and microscopic deterioration of gut after intestinal ischemia/reperfusion

**Figure 1:**
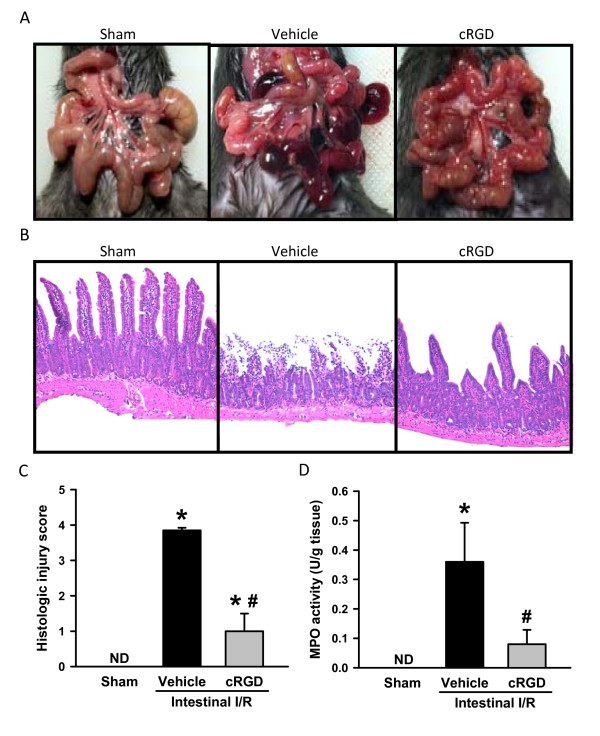
**cRGD attenuates gut damage and microscopic deterioration of gut after intestinal ischemia/reperfusion**. Mice treated with normal saline or cyclic arginine-glycine-aspartate peptide (cRGD) were subjected to intestinal ischemia/reperfusion (I/R). After 4 hours, mice were euthanized. (**A**) Representative images for the gross morphological appearance of the gut from sham, vehicle, and cRGD treatment groups. (**B**) Gut tissues harvested 4 hours after intestinal I/R were stained with H & E, and examined under light microscopy at 200× magnification. Representative images for sham, vehicle, and cRGD treatment groups. (**C) **Histologic injury scores of the gut in different groups were quantified as described in Materials and methods. (**D**) Gut tissue myeloperoxidase (MPO) activity was determined spectrophotometrically. Data expressed as mean ± standard error (*n *= 5/group). *P < 0.05 versus sham, ^#^*P *< 0.05 versus vehicle. ND, nondetectable.

The gut exhibited remarkably severe inflammation, necrosis, and ischemic darkness with vascular congestion all over the tissue after intestinal I/R, compared with the sham group (Figure 1A). Following treatment with cRGD, the severity of gut damage was improved to only have a minor sign of necrosis and ischemic congestion, although the gut still remained moderately edematous (Figure 1A). On examination of the histopathological changes, severe mucosal damage with denudation of villi and collapse of small vessels was microscopically observed in the gut tissue after I/R in comparison with the sham group (Figure 1B). The integrity of morphological structure and height of the villi were well preserved in the gut of cRGD-treated animals when compared with the vehicle ones (Figure 1B). As quantified in Figure 1C, animals undergoing I/R with vehicle treatment exhibited a significant increase in the gut histologic injury score when compared with the sham animals, which was reduced by 70.6% with administration of cRGD. MPO activity, an indicator of neutrophil content, in gut tissue rose from nondetectable in the sham group to 0.36 ± 0.13 U/g tissue after I/R (Figure 1D). However, administration of cRGD resulted in a 77.8% inhibition of MPO activity in the gut tissue after I/R (Figure 1D).

### cRGD improves microscopic deterioration of the lungs after intestinal ischemia/reperfusion

**Figure 2:**
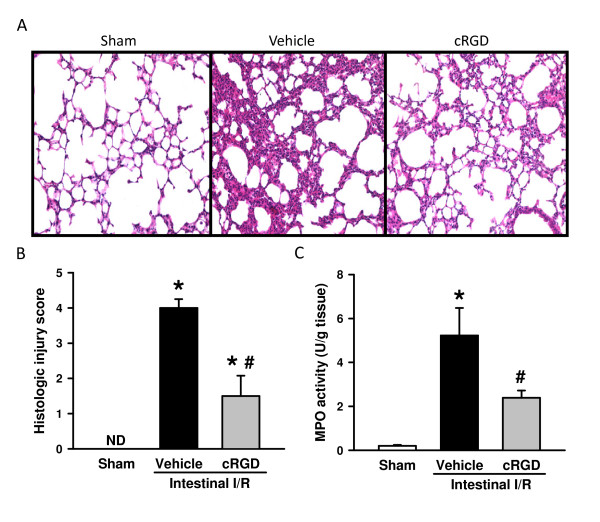
**cRGD improves microscopic deterioration of the lungs after intestinal ischemia/reperfusion**. (**A**) Lung tissues harvested 4 hours after intestinal ischemia/reperfusion (I/R) were stained with H & E, and examined under light microscopy at 200× magnification. Representative images for sham, vehicle, and cyclic arginine-glycine-aspartate peptide (cRGD) treatment groups. (**B) **Histologic injury scores of the lungs in different groups were quantified as described in Materials and methods. (**C**) Lung tissue myeloperoxidase (MPO) activity was determined spectrophotometrically. Data expressed as mean ± standard error (*n *= 5/group). *P < 0.05 versus sham, ^#^*P *< 0.05 versus vehicle. ND, nondetectable.

The lung is regarded as the most vulnerable remote organ injured after intestinal I/R. The lung tissues after I/R presented substantial morphological changes, including alveolar collapse, edema, hemorrhage and infiltration of inflammatory cells in comparison with the sham group (Figure 2A). In contrast, administration of cRGD dramatically reduced microscopic deterioration in comparison with the vehicle group (Figure 2A). As quantified in Figure 2B, mice undergoing I/R with vehicle treatment exhibited a significant increase in the lung histologic injury score when compared with the sham animals, which was reduced by 60.0% with administration of cRGD. MPO activity in the lungs was increased after intestinal I/R, whereas administration of cRGD significantly attenuated lung MPO activity by 54.2% (Figure 2C).

### Expression of adhesion molecules for neutrophil infiltration is upregulated after intestinal ischemia/reperfusion

**Figure 3:**
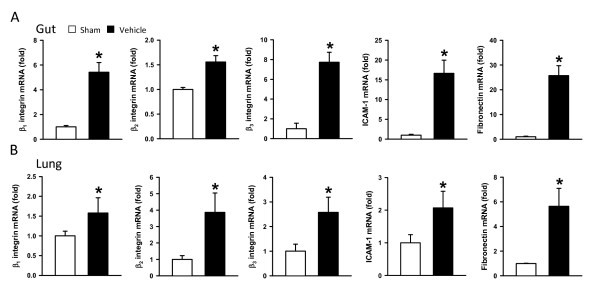
**Expression of adhesion molecules for neutrophil infiltration is upregulated after ischemia/reperfusion**. Gut and lung tissues were harvested 4 hours after intestinal ischemia/reperfusion (I/R). The mRNA levels of β_1_, β_2_, and β_3 _integrins, intercellular adhesion molecule-1 (ICAM-1), and fibronectin in the (**A**) gut and (**B**) lungs were determined by real-time RT-PCR analysis. Their expression levels were normalized to β-actin. The value of the sham group is designated 1 for comparison. Data expressed as mean ± standard error (*n *= 5/group). *P < 0.05 versus sham.

Integrins are the major surface receptors that mediate the interaction of neutrophils to other cells and ECM. ICAM-1 expressed on endothelial cells and fibronectin, an ECM protein, bind to integrins to regulate neutrophil infiltration. The change of mRNA expression of β_1_, β_2 _and β_3 _integrins, ICAM-1 and fibronectin in the tissues after intestinal I/R was determined by real-time RT-PCR analysis. In the gut, expression levels of these three integrins and two ligands were significantly increased after I/R, ranging from 1.7-fold to 25.7-fold in comparison with the sham group (Figure 3A). Similarly, in the lungs, expression levels of these five proteins had a 1.6-fold to 5.4-fold increase in the vehicle in comparison with the sham group (Figure 3B). The observation of the upregulation of integrins and their ligands indicates an enhanced interaction of neutrophils with the endothelium and ECM, leading to infiltration after intestinal I/R.

### cRGD inhibits neutrophils and macrophages infiltrating the lungs after intestinal ischemia/reperfusion

**Figure 4:**
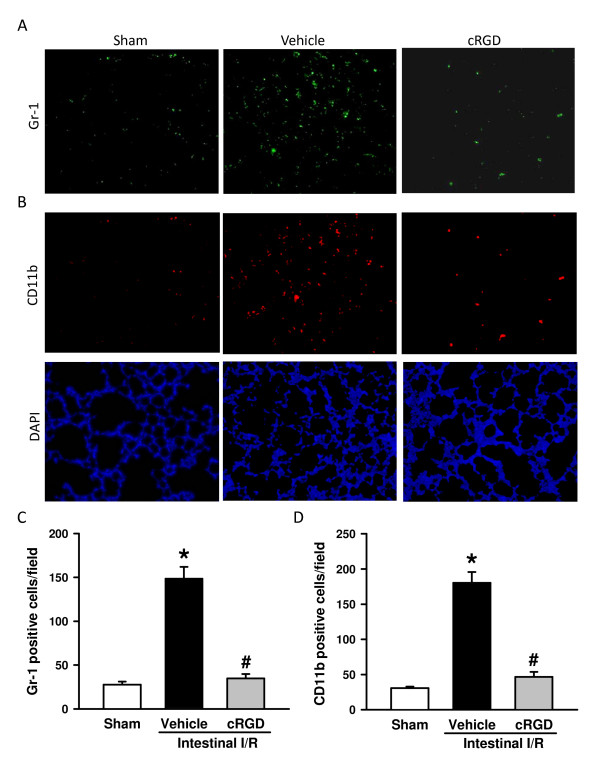
**cRGD inhibits neutrophils and macrophages infiltrating the lungs after intestinal ischemia/reperfusion**. Lung tissues harvested 4 hours after intestinal ischemia/reperfusion (I/R) were sectioned and immunostained with (**A**) Gr-1 (green) for detecting neutrophils and (**B**) CD11b (red, upper panel) for detecting macrophages. Lower panels: 4',6-diamidino-2-phenylindole (DAPI; blue) staining for nucleus. Representative images for sham, vehicle, and cRGD treatment groups at 200× magnification. Graphical representation of (**C**) Gr-1-positive and (**D**) CD11b-positive staining cells averaged over 10 microscopic fields per animal. Data expressed as mean ± standard error (*n *= 5/group). *P < 0.05 versus sham, ^#^*P *< 0.05 versus vehicle. cRGD, cyclic arginine-glycine-aspartate peptide.

To verify the effect of cRGD treatment on neutrophil infiltration, the lung tissues were immunostained with anti-Gr-1 antibodies. As shown in Figure 4A, C, the I/R resulted in an increase of the number of Gr-1-positive cells from 27.7 ± 3.5 to 145.0 ± 16.1 cells/field, whereas with the treatment of cRGD the number of these cells significantly reduced to 38.0 ± 6.7 cells/field. The lung tissues were also immunostained with anti-CD11b antibodies to detect the infiltrated macrophages. Similarly, the number of CD11b-positive cells in the sham, vehicle, and cRGD treatment groups was 30.7 ± 2.2, 180.3 ± 15.5, and 46.7 ± 7.2 cells/field, respectively (Figure 4B, D). cRGD treatment thus effectively prevents the neutrophils and macrophages infiltrating the lungs after intestinal I/R. We also stained the gut tissues with anti-Gr-1 and anti-CD11b antibodies. Although a reduction of neutrophils and macrophages was observed in the gut of the cRGD-treated group, the number of stained cells was very low and sparse (data not shown).

### cRGD lowers the inflammatory response after intestinal ischemia/reperfusion

**Figure 5:**
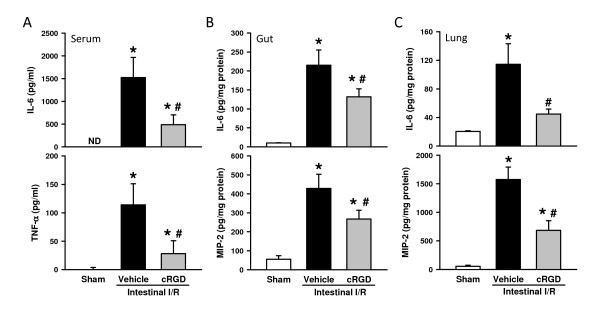
**cRGD lowers the inflammatory response after intestinal ischemia/reperfusion**. Blood and tissues were harvested 4 hours after intestinal ischemia/reperfusion (I/R). (**A**) Serum levels of IL-6 and TNF-α were determined by ELISA. **(B) **Gut and (**C**) lung levels of IL-6 and macrophage inflammatory protein-2 (MIP-2) were determined by ELISA. Data expressed as mean ± standard error (*n *= 5/group). *P < 0.05 versus sham, ^#^*P *< 0.05 versus vehicle. ND, nondetectable. cRGD, cyclic arginine-glycine-aspartate peptide.

Excessive elevation of proinflammatory cytokines is a major contributor in remote organ injury after intestinal I/R. Serum levels of proinflammatory cytokines TNF-α and IL-6 were increased to 102.0 ± 30.3 and 1,524.1 ± 493.0 pg/ml after I/R, respectively, while their levels are not detectable in the sham group (Figure 5A). Serum levels of TNF-α and IL-6 decreased by 71.0 and 67.9% with cRGD treatment, respectively, in comparison with the vehicle group (Figure 5A). After intestinal I/R, IL-6 and chemokine MIP-2 were increased in the gut tissues, while IL-6 and MIP-2 levels in the gut treated with cRGD were reduced by 38.6 and 24.4%, respectively, in comparison with the vehicle group (Figure 5B). Similarly, the elevation of IL-6 and MIP-2 levels in the lungs after I/R was inhibited by 60.8% and 56.7% with cRGD treatment, respectively (Figure 5C).

### cRGD inhibits apoptosis in the lungs after intestinal ischemia/reperfusion

**Figure 6:**
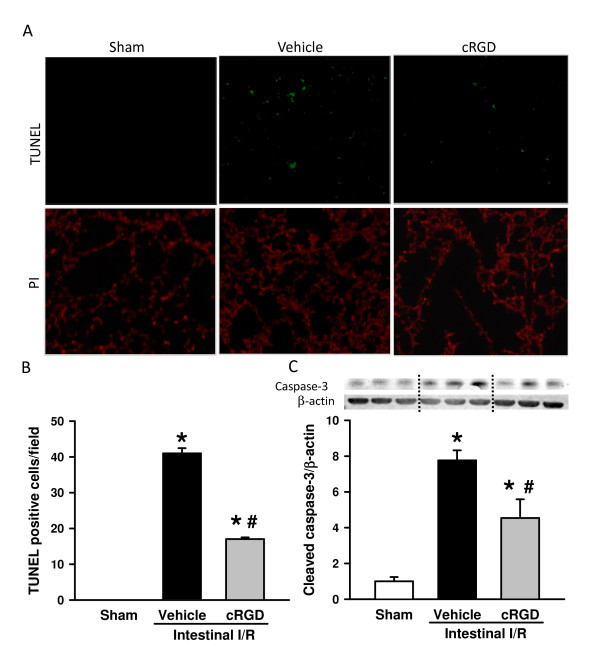
**cRGD inhibits apoptosis in the lungs after intestinal ischemia/reperfusion**. Lung tissues were harvested 4 hours after intestinal ischemia/reperfusion (I/R). **(A) **Lung sections were subjected to terminal deoxynucleotidyl transferase dUTP nick end-labeling (TUNEL) staining. Representative images for sham, vehicle, and cRGD treatment groups at 200× magnification. Upper panel: TUNEL-positive (green); lower panel: propidium iodide (PI; red) staining for nucleus. (**B**) Graphical representation of TUNEL-positive staining cells averaged over 10 microscopic fields per animal. (**C**) Levels of cleaved caspase-3 in the lung tissues determined by western blotting. Representative blots against cleaved caspase-3 and β-actin. Blots were scanned and quantified with densitometry. The levels of protein expression in the sham group are designated 1 for comparison. Data are expressed as mean ± standard error (*n *= 5/group). *P < 0.05 versus sham, ^#^*P *< 0.05 versus vehicle. cRGD, cyclic arginine-glycine-aspartate peptide.

To determine the effect of cRGD treatment on apoptosis, a TUNEL assay, a common method for detecting DNA fragmentation, was conducted immunohistochemically in the lung tissues. As shown in Figure 6A, B, the apoptotic cells in the lungs were elevated from nondetectable to well observed after intestinal I/R. Treatment of cRGD significantly reduced the number of apoptotic cells by 59.5% in the lungs in comparison with the vehicle group (Figure 6A, B). In addition, the levels of cleaved caspase-3, another marker of cell apoptosis, determined by Western blotting, were increased in the lungs of the vehicle group in comparison with those in the sham group (Figure 6C). Again, administration of cRGD significantly reduced the levels of cleaved caspase-3 after intestinal I/R by 57.0% (Figure 6C).

## Discussion

The lung is the most susceptible remote organ to be injured after intestinal I/R, which makes ALI a major cause for death [8,9,11]. Migration of activated neutrophils into the lungs is a hallmark for the progression of ALI [10], which is mainly mediated through the integrin-dependent pathway [17]. Synthetic peptides containing the RGD sequence have been demonstrated to be able to disrupt the cell-cell and cell-matrix interaction mediated by integrins, as well as to inhibit the cellular activity stimulated by integrin engagement.

In the current study, we showed that treatment with cRGD improved the integrity of tissue morphology not only locally on the gut but also remotely on the lungs after intestinal I/R. cRGD treatment effectively inhibited neutrophil infiltration to the organs, demonstrated by the reduction of MPO activity in the gut and lungs, and the number of Gr-1-positive cells in the lungs. cRGD treatment also attenuated the inflammatory response by decreasing the levels of TNF-α and IL-6 in serum, as well as IL-6 and MIP-2 in the gut and lungs. Correspondingly, the number of macrophages stained with CD11b in the lungs was reduced. Finally, the lungs of the cRGD-treated animals had less apoptotic cells, demonstrated by TUNEL staining and detection of cleaved caspase-3.

To our knowledge, this is the first study showing a protective effect of cRGD on ALI induced by intestinal I/R, although its effectiveness has also been demonstrated on ameliorating the injury in other organs that had undergone I/R [30,31]. In steatotic liver cold ischemia followed by transplantation, administration of a cyclic RGD peptide (CRGDGWC) inhibited the recruitment of macrophages and neutrophils, as well as depressed the expression of proinflammatory mediators, inducible nitric oxide synthase and IFN-γ [30]. In addition, the expression of matrix metaloproteinase-9, a gelatinase involved in leukocyte migration within the damaged liver was inhibited by the treatment [30]. In a rat model of renal I/R, cyclic RGD peptides (RGDDFV and RGDDFLG) effectively ameliorate acute renal failure [31]. Both peptides were effective at lowering the tubular obstruction by predominantly preventing cell-cell and cell-matrix adhesions [31]. Cyclic RGDFV had a higher potential than cyclic RGDFLG in inhibiting cell-matrix adhesion, but both peptides were equipotent in disrupting cell-cell adhesion [31]. The relative affinity and specificity of the RGD peptides to other proteins have been indicated to be affected by other amino acid residues flanking the RGD motif [35]. Cyclic CRGDGWC has higher affinity for the α_5_β_1 _integrin [36], while cyclic RGDFV more selectively interacts with α_v_β_3 _and α_v_β_5 _integrins [32].

RGD peptides can be synthesized in two different structure types of linear and cyclic formats. Cyclic RGD is much more stable than that of linear RGD under normal conditions [37]. Linear RGD, such as GRGDS and GRGDNP, are rapidly metabolized *in vivo *and have a relatively low affinity for integrins [37]. On the contrary, linear RGD is more flexible in comparison with cyclic RGD. However, the cell permeability of linear RGD is concerning. Linear RGD induces apoptosis without any requirement for integrin-mediated cell clustering or signals. Instead, linear RGD enters cells to bind pro-caspase-3 containing a potential RGD-binding motif (aspartate-aspartate-methionine), resulting in activation of caspase-3 [38]. In contrast, our study with the treatment of cRGD showed a reduction of apoptotic cells in the lungs after intestinal I/R, suggesting that very limited cRGD in the cells interacted with pro-caspasae-3. This difference may be due to the rigid structure of cyclic peptides in comparison with the linear structures.

The mechanism of the cRGD on alleviating ALI-induced by intestinal I/R is attributed to the inhibition of neutrophils infiltrating the lungs. Over-recruitment of activated neutrophils causes excessive release of proteolytic enzymes, such as elastase and MPO, and reactive oxygen species including hydrogen peroxide and superoxide. Excessive production of these molecules causes disruption of endothelial barrier functions and promotes extravascular host tissue damage [19,39]. Furthermore, neutrophils can produce cytokines and chemokines that enhance the acute inflammatory response [40]. Using real-time RT-PCR analysis, we detected an increased expression of several adhesive molecules, including β_1_, β_2_, and β_3 _integrins, ICAM-1 and fibronectin, which are all involved in neutrophil transmigration after intestinal I/R. This observation further supports the rationale of targeting integrin-mediated interaction to control neutrophil infiltration into the organs.

In addition to neutrophils, a reduction of macrophages in the lungs was also observed in the cRGD-treated animals. Macrophages are the key cell types involved in the production of proinflammatory cytokines such as TNF-α and IL-6, as well as chemokines including monocyte chemoattractant protein-1, and MIP-2 [41]. MIP-2, a murine analog of IL-8, is a potent chemoattractant for neutrophil recruitment and activation [42]. By depletion of alveolar macrophages, alveolar macrophages have been indicated to play an essential role in ALI induced by intestinal and acute lung I/R [11,20]. Furthermore, a recent study demonstrated that cyclic RGD could attenuate the TNF-α secretion from macrophages by inhibiting the ligation of α_v_β_3 _integrin for NF-κB activation [43].

In this study, administration of cRGD at 5 mg/kg BW was based on a recent report showing that the linear RGDS peptide at 5 mg/kg BW significantly inhibited neutrophils infiltrating the lungs in a lipopolysaccharide-induced ALI model [29]. In addition, cyclic RGD at 4 mg/kg BW had been applied in the study of steatotic liver cold ischemia and reperfusion injury [30]. We also performed a pilot study at a dose of 10 mg/kg BW cRGD, but did not observe a noticeable difference in comparison with the 5 mg/kg BW dose. While excessive neutrophil infiltration causes organ damage, neutrophils are necessary for the killing of invaded pathogens [19,39]. We therefore speculate that administration of cRGD at increasingly high doses may lessen its protective effect on ALI. To evaluate the overall effect of cRGD on attenuating organ injury and inhibiting the inflammatory response, administration of normal saline (vehicle) was used as a baseline for the comparison. Any random peptide sequence used as control could incur potential unidentified off-target effects. Moreover, the surgical procedure of intestinal I/R described here is a severe model. Most mice died within 20 hours after I/R in our pilot study, which makes it very difficult to study the effect of cRGD at later time points. In essence, therefore, the focus of this study was to examine the onset of ALI and monitor the underlying immunologic mechanisms at the early time points.

## Conclusions

ALI is a common complication that occurs after intestinal I/R and contributes to its high mortality rate. Excessive neutrophil infiltration into the lungs is a hallmark of ALI. Integrin-mediated interaction plays an important role in regulating neutrophil transmigration as well as activating various cellular activities. Administration of cRGD attenuated the severity of ALI and local gut injury in mice after intestinal I/R. The numbers of neutrophils and macrophages infiltrating the lungs remarkably decreased after treatment. In addition, the levels of proinflammatory cytokines and chemokines in the circulation and organs were suppressed by cRGD treatment. Furthermore, the induction of apoptosis in the lungs after I/R was inhibited by cRGD. These results indicate that cRGD has the potential to develop as an effective therapeutic agent for treating ALI induced by intestinal I/R.

## Key messages

• ALI is a common complication with high mortality in humans, and neutrophil infiltration to the lungs is a crucial factor for the progression of ALI.

• Integrin-mediated interaction significantly contributes to neutrophil transmigration and activation of various cellular activities, which can be inhibited by the peptides containing the RGD sequence.

• Treatment with cRGD attenuates ALI and gut injury, inhibits neutrophils and macrophages infiltrating the lungs, suppresses the levels of proinflammatory cytokines and chemokines, and reduces apoptosis in mice subjected to intestinal I/R.

• cRGD has a potential to develop as a therapeutic agent for treating patients suffering from ALI.

## Abbreviations

ALI: acute lung injury; BW: body weight; cRGD: cyclic arginine-glycine-aspartate peptide (cyclo RGDfV); ECM: extracellular matrix; ELISA: enzyme-linked immunosorbent assay; H & E: hematoxylin and eosin; ICAM: intercellular adhesion molecule; IL: interleukin; I/R: ischemia/reperfusion; MIP-2: macrophage inflammatory protein-2; MPO: myeloperoxidase; PBS: phosphate-buffered saline; PCR: polymerase chain reaction; RGD: arginine-glycine-aspartate; RT: reverse transcriptase; TNF: tumor necrosis factor; TUNEL: terminal deoxynucleotidyl transferase dUTP nick end-labeling.

## Competing interests

The authors declare that they have no competing interests.

## Authors' contributions

SM carried out all animal experiments, performed biochemical measurements, analyzed the data, conducted the statistical analysis, and drafted the manuscript. W-LY initiated the project, designed the experiments, assisted in data analysis and interpretation, and revised the manuscript for the revised submission. MA participated in its design and performed experiments. AJ participated in analysis and interpretation of data, and critically revised the manuscript. PW conceived of the study, participated in analysis and interpretation of data, and critically reviewed and approved the manuscript. All authors read and approved the final manuscript.
